# *Proechimys* (Rodentia, Echimyidae): characterization and taxonomic considerations of a form with a very low diploid number and a multiple sex chromosome system

**DOI:** 10.1186/1471-2156-14-21

**Published:** 2013-03-11

**Authors:** Paulo JS Amaral, Cleusa Y Nagamachi, Renata CR Noronha, Marlyson JR Costa, Adenilson L Pereira, Rogerio V Rossi, Ana C Mendes-Oliveira, Julio C Pieczarka

**Affiliations:** 1 Laboratório de Citogenética, Instituto de Ciências Biológicas, Universidade Federal do Pará, Campus do Guamá, Av. Bernardo Sayão, sn. Guamá, Belém – Pará, 66075-900, Brazil; 2 CNPq Researcher, Belém – Pará, Brazil; 3 FAPESPA Doctoral scholarship in Genetics and Molecular Biology, Belém – Pará, Brazil; 4 CAPES Master scholarship in Genetics and Molecular Biology, Belém – Pará, Brazil; 5 Departamento de Biologia e Zoologia, IB-UFMT, Belém – Pará, Brazil; 6 Laboratório de Zoologia de Vertebrados, ICB, UFPA, Belém – Pará, Brazil; 7 PIBIC-UFPA scholarship, Belém – Pará, Brazil

**Keywords:** Rodent, *Proechimys*, Cytogenetics, Chromosomal evolution, Multiple sex system, FISH

## Abstract

**Background:**

*Proechimys* is the most diverse genus in family Echimyidae, comprising 25 species (two of which are polytypic) and 39 taxa. Despite the numerous forms of this rodent and their abundance in nature, there are many taxonomic problems due to phenotypic similarities within the genus and high intraspecific variation. Extensive karyotypic variation has been noted, however, with diploid numbers (2n) ranging from 14 to 62 chromosomes. Some heteromorphism can be found, and 57 different karyotypes have been described to date.

**Results:**

In the present work, we describe a cytotype with a very low 2n. Specimens of *Proechimys* cf. *longicaudatus* were collected from two different places in northern Mato Grosso state, Brazil (12°54″S, 52°22″W and 9°51′17″S, 58°14′53″W). The females and males had 16 and 17 chromosomes, respectively; all chromosomes were acrocentric, with the exception of the X chromosome, which was bi-armed. The sex chromosome system was found to be XY_1_Y_2_, originating from a Robertsonian rearrangement involving the X and a large acrocentric autosome. Females had two Neo-X chromosomes, and males had one Neo-X and two Y chromosomes. NOR staining was found in the interstitial region of one autosomal pair.

**Conclusions:**

Comparison of this karyotype with those described in the literature revealed that *Proechimys* with similar karyotypes had previously been collected from nearby localities. We therefore suggest that this *Proechimys* belongs to a different taxon, and is either a new species or one that requires reassessment.

## Background

The genus *Proechimys* is the most abundant among the non-volant mammals in Neotropical forests. It is found in lowland rainforests and is frequently represented by four or more sympatric species in mammalian communities [1]. Twenty-five species have been recognized in the genus [2], two of which are polytypic, for a total of 39 taxa in the species group. The most comprehensive taxonomic review of the genus [3] used craniodental and bacular traits to assign all of the nominal taxa to nine species groups, as follows: *guyannensis* (10 nominal taxa), *goeldii* (11), *longicaudatus* (9), *simonsi* (3), *cuvieri* (1), *trinitatus* (9), *semispinosus* (13), *canicollis* (1), and *decumanus* (1). The geographic distribution of the *longicaudatus* group ranges from west of the Amazon basin in southern Colombia to north of the Paraná basin in northern Paraguay, in sympatry with the *goeldii*, *cuvieri* and *simonsi* groups. Due to a high level of morphological variation within populations and phenotypic similarities among the species of *Proechimys*, specimens are frequently identified at the species-group level, following the classification of Patton [3]. We do not yet have a conclusive phylogeny for the genus, making it difficult to understand the relationships among the species [4].

Cytogenetic studies offer a useful tool for understanding the evolution of this genus. The reported diploid numbers (2n) of *Proechimys* range from 14 to 62 chromosomes [5-9]. Initial chromosomal studies identified 13 cytotypes for this genus [6,10], a subsequent study recognized 28 karyotypes in 25 species [11], and an even later report identified 52 karyotypes [8]. Most recently, five more karyotypes were added for a current total of 57 karyotypes in *Proechimys*[9,12].

**Figure 1:**
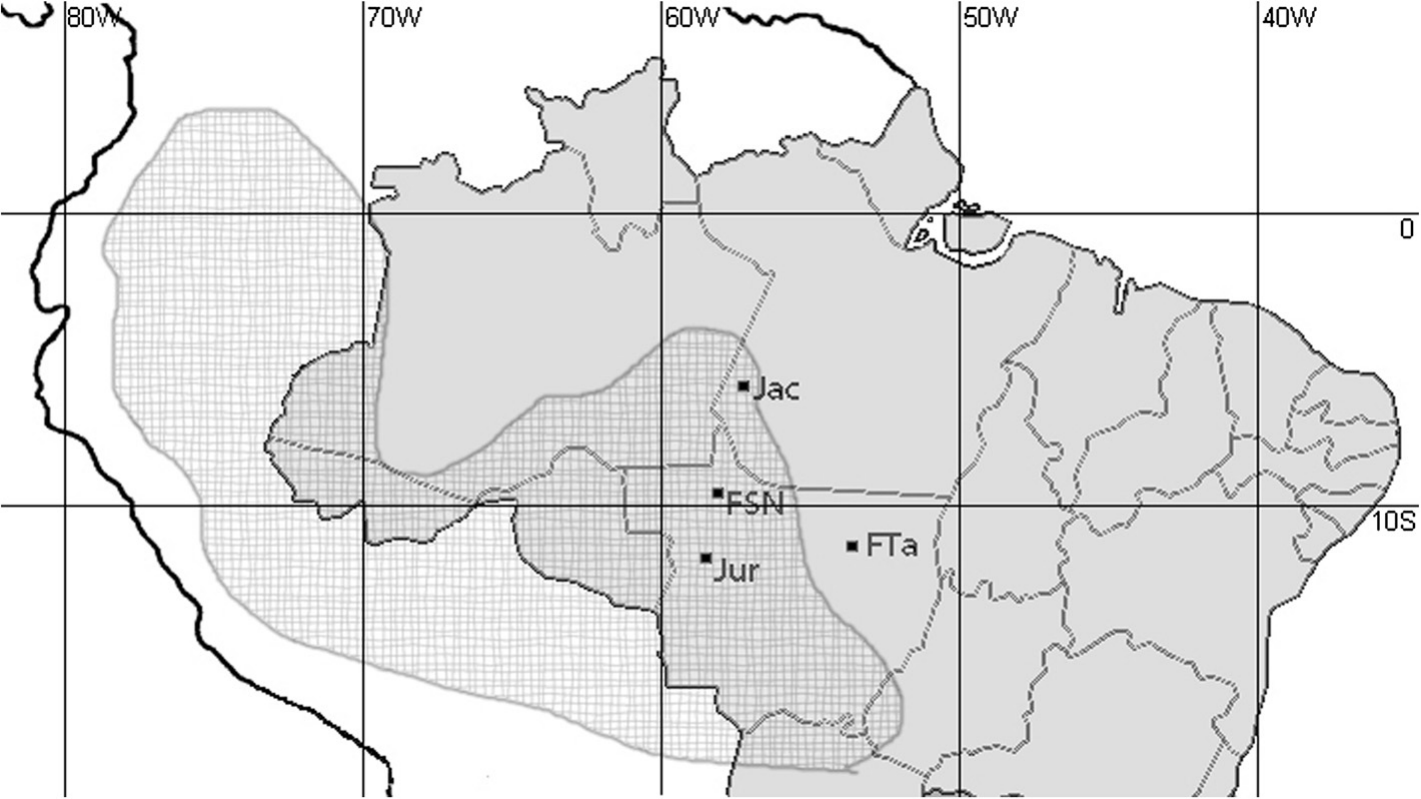
**Geographic locations from which *Proechimys *samples were collected for the present study and previous work involving karyotypes with low diploid numbers.** Abbreviations: FTa, Tanguro Farm; FSMN, São Nicolau Farm; Jac, Jacaréacanga-Flexal [5]; and Jur, Juruena [12]. The checkered area shows the geographic distribution of *longicaudatus*[13].

The 2n of some species within the *Proechimys* complex have recently been studied in more detail. Amaral *et al.* (unpublished data) reported *P. longicaudatus* with 2n = 28-30 (Table 1) and *goeldii* with 2n = 24-28. Although these ranges overlapped, differences in chromosome morphology created karyotypic differences that were reflected in the Fundamental Numbers (FN) of 40 to 44 in *goeldii* and 14 to 52 in *longicaudatus*. However, there have been descriptions of divergent karyotypes characterized by reduced 2n in the southern Amazonia, where species of the *cuvieri*, *goeldii*, *guyannensis*, and *longicaudatus* groups can occur [3,13] within the geographic distribution of the *Proechimys* species groups. For example, researchers reported karyotypes with 2n = 14 to 17 for an unidentified species in Jacaréacanga-Flexal, Pará, Brazil (6°16^′^48″S; 57°39^′^04″W) [5], and a recent report described a species of the *goeldii* group with 2n = 15 from a more southern location in Juruena, Mato Grosso (12°51^′^31″S; 58°55^′^08″W) [12] (Figure 1). Although the latter sample was described as *goeldii*[12], given the difficulties in identifying the *Proechimys* species groups, we questioned which group (*cuvieri*, *goeldii*, *guyannensis*, or *longicaudatus*) was actually represented by these low diploid-number rodents. To answer this question, we collected samples of *Proechimys* from this region, analyzed their karyotypes, diploid numbers and morphological traits, and sought to assign their group(s).

**Table 1:** **Karyotypes of species from group *longicaudatus *from literature and present work (2n = diploid number; FN = Fundamental Number; M-male; F- female)**

	**2n**	**FN**	**Geographic location**	**Reference**
**Taxon**				
*P. gularis*^a^	30(M)	48	Limoncocha (Napo, Equador)	*
*P. brevicauda*	28-30(M/F)	48-50	South of Peru	*
*P. brevicauda*	28(M/F)	48	Juruá river (Acre, Brazil)	***
*P. brevicauda*	30(M)	48	Cenepa river (Amazonas and Peru)	*****
*P. longicaudatus*	28(M/F)	46	Right side, Madeira river (Amazonas, Brazil)	**
*P. longicaudatus*	30(F)	52	Jamari river (Rondonia, Brazil), Juruena and Aripuanã (Mato Grosso, Brazil)	****
*P. longicaudatus*	28(M/F)	48	Samuel Hydroelectric reservoir, Jamari river (08°45^′^ S,63°26^′^ W), Rondônia, Brazil	****
*P. longicaudatus*	28(M/F)	50	Apiacás (09°34^′^ S, 57°23^′^ W), Mato Grosso-MT, Brazil	****
*P. longicaudatus*	28(M)	50	Emas National Park (18°15^′^ S, 52°53^′^ W), Goiás, Brazil	****
*P.* gr. *longicaudatus*	17(M)	14	Tanguro farm, Querência, MT, Brazil (12° 54″S; 52° 22″W); São Nicolau Farm, Cotriguaçu-MT, Brazil (S09°51^′^17.6″/W058°14^′^53.8″//S09°49^′^24.9″/W058°15^′^32.0″).	Present work
*P.* gr. *longicaudatus*	16(F)	14	Tanguro farm, Querência, MT, Brazil (12° 54″S; 52° 22″W); São Nicolau Farm, Cotriguaçu-MT, Brazil (S09°51^′^17.6″/W058°14^′^53.8″//S09°49^′^24.9″/W058°15^′^32.0″).	Present work

References: *[8]; **Ribeiro (2006 *unpublished data*); ***[4]; ****[12]; *****[13].

^a^ Species understood as synonymous to *P. brevicauda*[2].

## Methods

We analyzed the karyotypes of seven specimens of *Proechimys* from south of the Amazon forest (Figure 1). One male and one female (Museu Paraense Emilio Goeldi, PA-Brazil, voucher numbers FT-118 and FT-119, respectively) were collected at Tanguro Farm, located near the district of Querência in the state of Mato Grosso, Brazil (12°54″S; 52°22″W). Two more males (University of Mato Grosso Museum, MT-Brazil, voucher numbers MSN-56 and MSN-57) and three females (voucher numbers MSN-151, MSN-152 and MSN-157) were collected at São Nicolau Farm, in the district of Cotriguaçu in the state of Mato Grosso, Brazil (9°51^′^17″S; 58°14^′^53″W) (Figure 1). The animals were collected specifically for research, using Tomahawk, Sherman and Pitfall traps. Our team has a permanent permit for collecting samples of Brazilian biodiversity (SISBIO 13248). The specimens were euthanized by carbon dioxide inhalation. Bone marrow samples were taken in the field, and chromosome spreads were processed in the laboratory. Mitotic and meiotic chromosomes were obtained as previously described [14,15]. Metaphases were analyzed by conventional staining, G-banding [16], C-banding [17] and Ag-NOR staining [18]. FISH with telomeric probes was performed using a commercially available kit (Oncor). Bright-field images were captured with an Olympus microscope and analyzed using the SpectraView software (Applied Spectral Imaging). Fluorescent images were captured on a Zeiss Axiophot microscope with a CCD camera (AxioCam MR Monochrome) controlled using the AxioVision 3.0 software (Zeiss).

Morphological analyses were performed based on the craniodental characteristics described by Patton [3].

## Results

**Figure 2:**
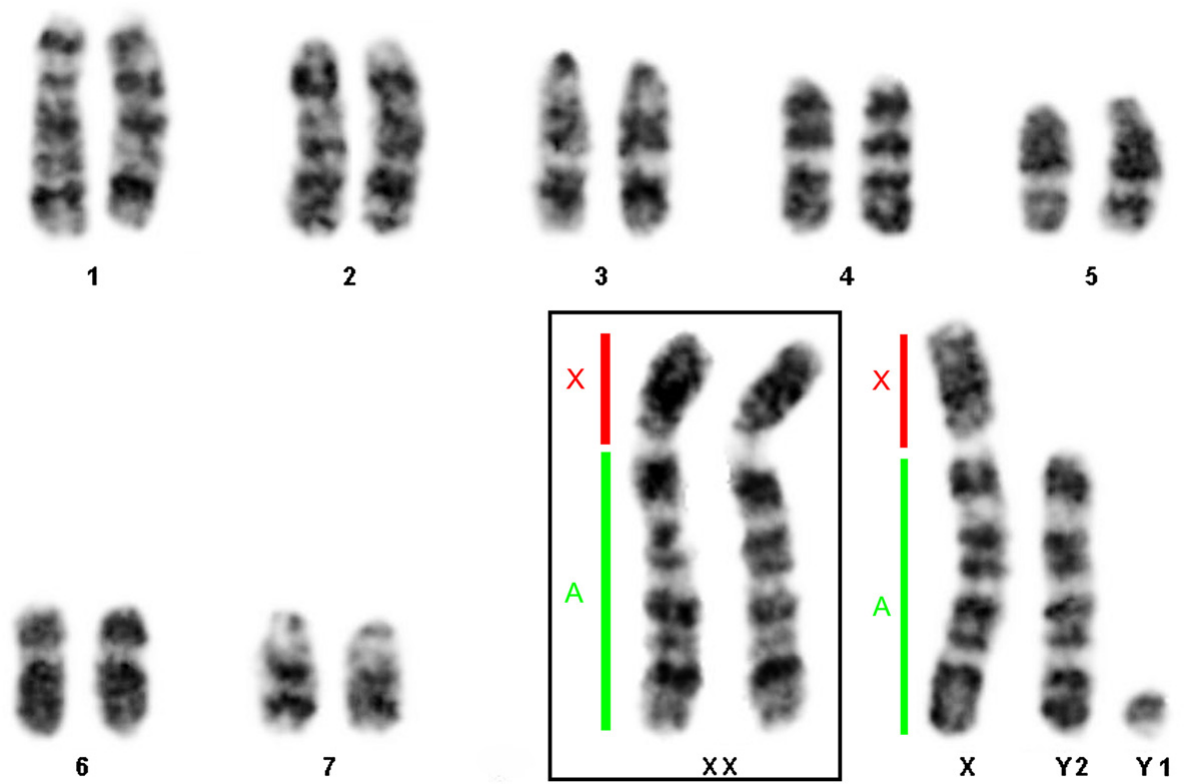
**G-banding pattern in a male *Proechimys *cf *. longicaudatus *.** For Neo-X, the X portion and the autosomal (A) portion are indicated. The box shows the XX female sex chromosomes.

**Figure 3:**
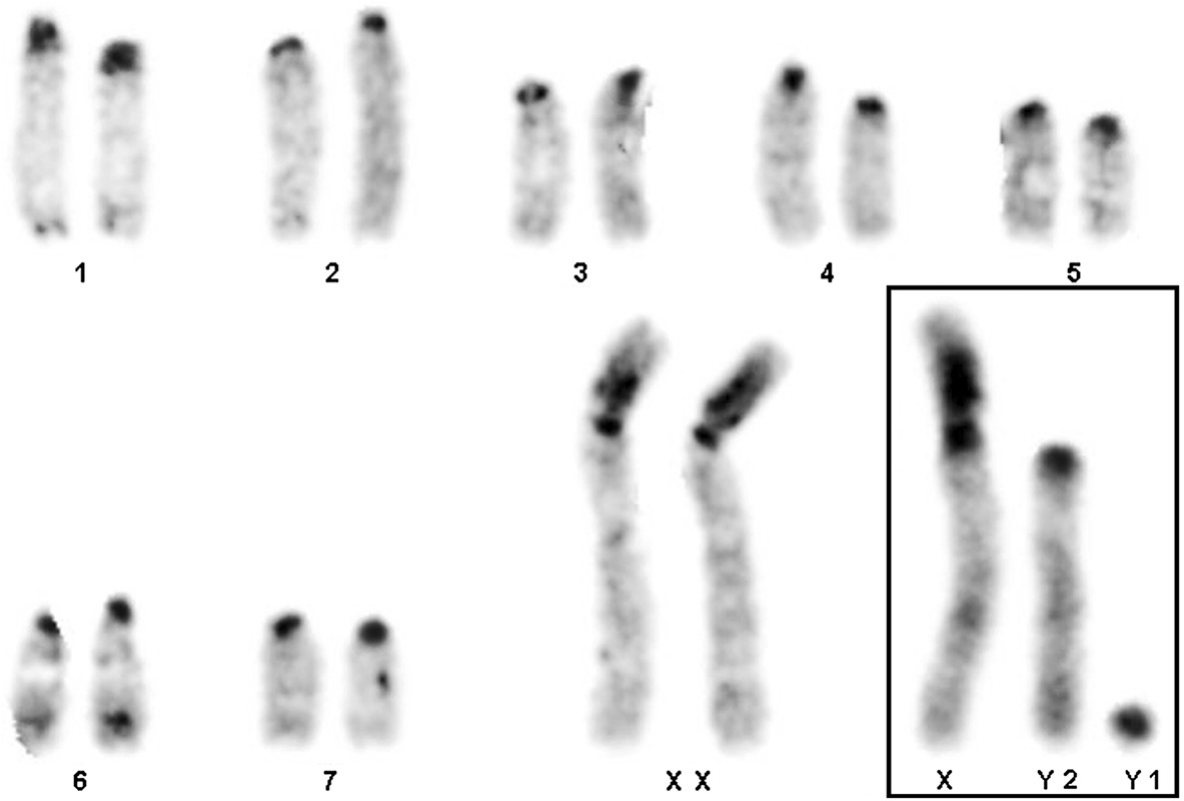
**C-banding pattern in a female *Proechimys *cf *. longicaudatus *.** The box shows the XY_1_Y_2_ = male sex chromosomes. All of the centromeric regions are C-banded.

**Figure 4:**
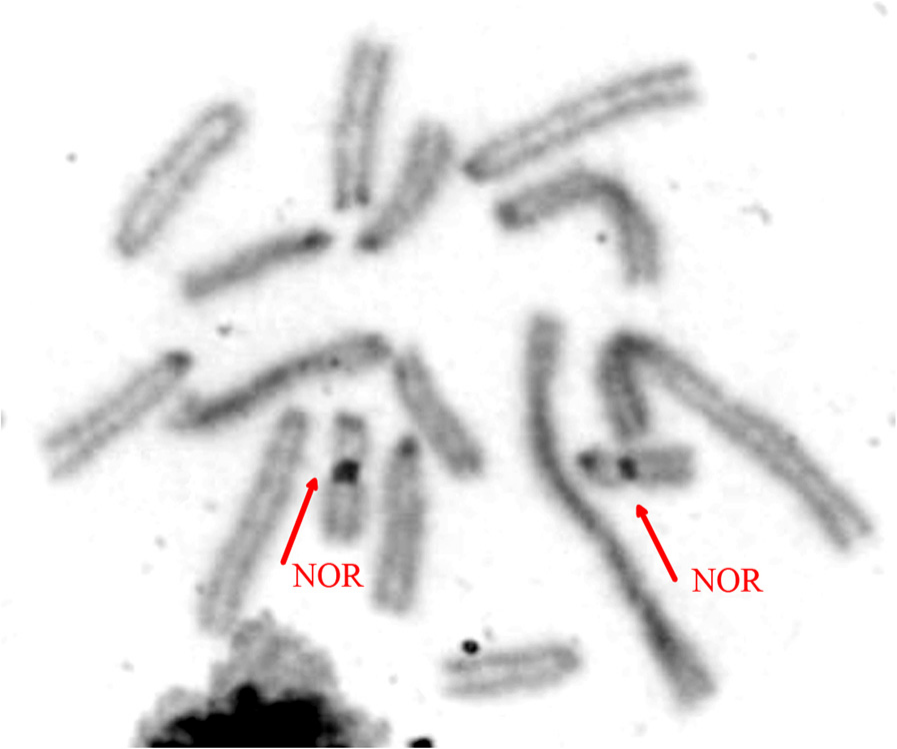
**NOR-staining in *Proechimys *cf. *longicaudatus*.** The arrows (red) shows the NOR staining in chromosome pair 6.

**Figure 5:**
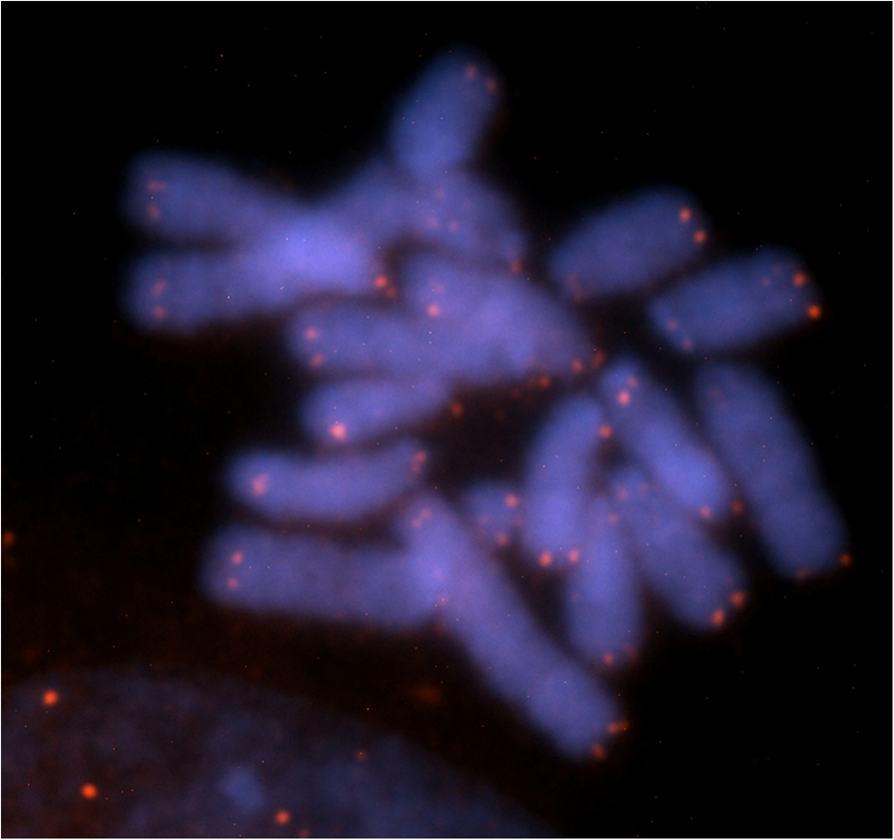
**FISH using human telomeric probes in *Proechimys *gr. *longicaudatus*.**

The males and females collected at Tanguro Farm had 2n = 17 and 16, respectively. All autosomes were acrocentric, the X chromosome was bi-armed, and the Y was the smallest acrocentric of the karyotype. The sex chromosome system was found to be XX/XY_1_Y_2_ (Figure 2). C-banding demonstrated the presence of constitutive heterochromatin (CH) in the centromeric regions of all chromosome pairs. The X had a CH block in the proximal portion of the short arm (Figure 3), and the Y_1_ was almost entirely heterochromatic (Figure 3). NOR staining with silver nitrate (Ag-NOR) showed positive staining at the interstitial region of pair 6 (Figure 4). Telomeric sequences were found in the distal portions of all chromosome pairs. We did not find any interstitial telomeric sequences (ITS) or centromeric signals (Figure 5). The samples from São Nicolau Farm had similar karyotypic constitutions, patterns, and distributions of G- and C-banding.

**Figure 6:**
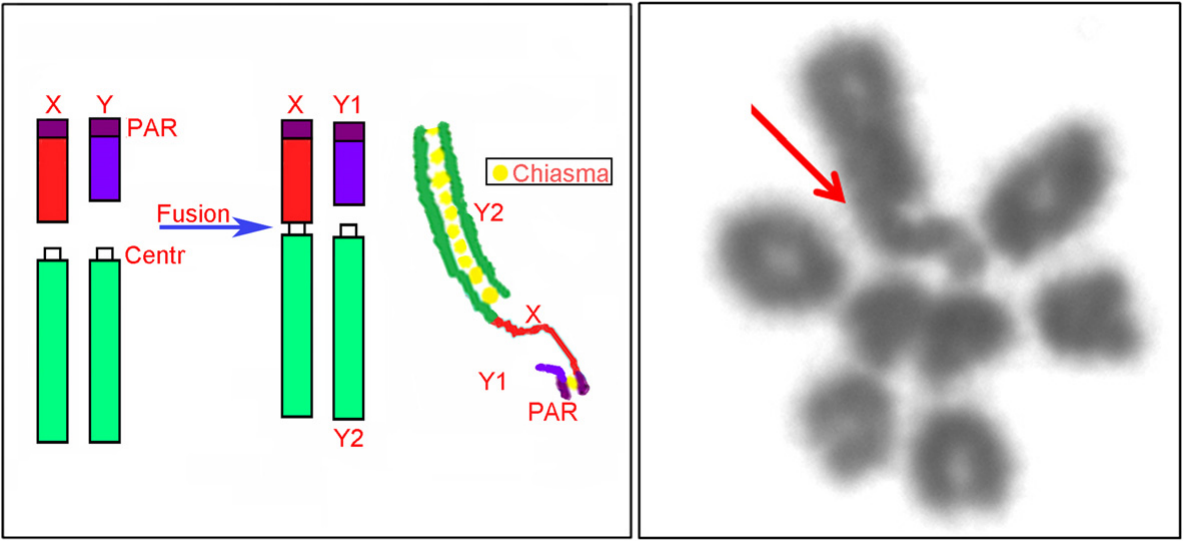
**Meiotic analysis of the XX/XY**_**1**_**Y**_**2 **_**system in *Proechimys *cf. *longicaudatus *Left: A diplotene in *Proechimys *cf *longicaudatus *with tip-to-tip pairing between the X and Y1, and a chiasma between X and Y2.** The red arrow shows the connection point between the X and the translocated autosome. Right: schematic of the rearrangement and chromosome pairing of the sex trivalent seen in the diplotene. Abbreviations: PAR, pseudo-autosomal region; Centr, centromere of the autosome; and A, autosome.

Meiotic analysis in diplotene–diakinesis revealed the presence of seven bivalents and a sex trivalent with two independent pairing regions: one between the original X and Y_1_ chromosomes (called the pseudoautosomal region, where the chromosomes linked at their tips); and one with the translocated segment, which corresponded to the X and its autosomal homolog (Y_2_) and confirmed the multiple sex chromosome system. During prophase I, we observed a sex body (SB) in the pairing region between the X and Y_1_. It appeared similar to that seen in the simple sex determination system during the stages of leptotene, zygotene and pachytene, with heteropycnosis of the SB (Figure 6). The pairing region between the autosomal segment translocated to the X and the free autosome (Y_2_) did not show any differentiating characteristics, and thus resembled that of the other free autosomes (Figure 6).

Morphologically, the studied specimens fulfilled the criteria for assignment to the *longicaudatus* group: they had a lyre-shaped incisive foramen with a strongly constricted posterior portion; a maxillary terminus deeply grooved into the anterior palate; strongly franged posterior margins; a septum with an expanded premaxillary portion, a well-developed and keeled maxillary portion, and a ventrally exposed vomerine portion; an underdeveloped temporal ridge; a weakly developed ventral canal of the infraorbital foramen; and three counterfolds in the second and third upper molars.

## Discussion

Our specimens had an uneven diploid number due to the presence of a multiple sex chromosome determination system (XX/XY_1_Y_2_) that arose via a Robertsonian rearrangement between the X chromosome and the largest acrocentric autosome, creating a Neo-X. The females had 2n = 16 with two Neo-X chromosomes, while the males had 2n = 17 with one Neo-X, one Y_1_ (true Y) and one Y_2_ (the homolog of the autosome translocated to the X). In *Proechimys*, translocations involving the sex chromosomes and autosomes are far less common than autosomal intrachromosomal rearrangements. Translocations involving sex chromosomes and autosomes may cause a new meiotic configuration, leading to low fertility among heterozygotes and creating post-zygotic barriers that can lead to chromosomal speciation [19,20]. Many cytotaxonomic differences between species have arisen from variations in the sex chromosomes, coming mainly from constitutive heterochromatin addition/deletion, inversions and translocations [21]. Members of genus *Proechimys* typically have a simple sex chromosome system [8]; thus, the simple system appears to be a symplesiomorphy, while the multiple sex chromosome system described herein is an autapomorphy.

We sought to assign our low-chromosome-number specimens to the appropriate species group of *Proechimys* (i.e., *cuvieri*, *goeldii*, *guyannensis* or *longicaudatus*). Based on morphological traits, we positively identified our specimens as belonging to the *longicaudatus* group. Notably, the specimens from Tanguro Farm represent an eastward extension of the previously recognized geographic distribution of the low-chromosome-number group of *Proechimys* (Figure 1).

Only two species are currently recognized in the *longicaudatus* group: *P. brevicauda* and *P. longicaudatus*[2]. Two cytotypes have been described for *longicaudatus*; both had 2n = 28 [12,13], and their karyotypes differed significantly from those described herein. The G-banding patterns were different from one another, and we found it difficult to compare the previous data with our present results. Future chromosome painting studies should allow us to define the precise homologies among these karyotypes. The C-banding patterns also did not allow for precise comparison of our results with the previous descriptions. In some of the *Proechimys* studied to date, the NORs have been found in the distal regions of the large arms of various pairs [12]. Here, we found the NOR in the interstitial region of the large arm of chromosome pair 6.

We compared our data with the previously published karyotypes for *Proechimys* showing diploid numbers close to those of our samples, and found that our results were similar to those described in two prior papers [5,12]. Our karyotypes were consistent with those described for *P.* gr*. goeldii* (2n = 15) [12], which had an identical Neo-X chromosome. In the previous paper, the authors recognized that their sample represented a female heterozygous for a fusion in the third autosome pair, explaining the uneven diploid number. Without the fusion, the 2n would be 16. The authors did not publish the G-banding for this specimen, but when we inverted their metaphase DAPI-banding image using Adobe Photoshop, the banding pattern was very similar to our G-banding results, confirming that the two karyotypes were quite similar. The previous authors studied only one female, however, and thus did not realize that their pair 1 was actually a Neo-X, as described herein. The chromosome they proposed as the X is homologous to pair 5 in the present work. Given the chromosomal similarities between our karyotypes and the 2n = 15 karyotype described in the previous paper [12], we propose that all *Proechimys* with this karyotype should be considered members of the *longicaudatus* group instead of the *goeldii* group. Paradoxically, when we analyzed the previously described specimen [12], which is deposited in the Museu de Zoologia da Universidade de São Paulo (São Paulo, Brazil), we concluded that it may indeed be morphologically associated with the *goeldii* group. In contrast, our specimens from Fazenda Tanguro were found to be morphologically associated with the *longicaudatus* group. Given the karyotypic similarities discussed above, we argue that the previously described specimen [12] is probably a composite of two different specimens and species, with the skin and skull belonging to a species of the *goeldii* group, while the karyotype belongs to a species of the *longicaudatus* group. This could be reasonably explained by a labeling mistake made in the field. If this is not the case, it would seem that either the previously described specimen represents a morphologically atypical individual of the *longicaudatus* group, or there are two karyotypically similar low-diploid-numbered species of *Proechimys* in the southern Amazon, one belonging to the *longicaudatus* group (our specimens) and another belonging to the *goeldii* group [12].

In the other previous work describing a low-diploid-numbered species of *Proechimys*, seven different karyotypes were described in 38 specimens of *Proechimys* sp. collected in the Para state, Brazil [5]. These rodents were classified into three apparent species (designated sp1, sp2 and sp3). Of them, sp3 from the Jacaréacanga-Flexal locality (Figure 1; 6°16^′^48″S, 57°39^′^04″W) had 2n = 14-17 and a chromosome pair 1 that was identical to the Neo-X described herein and mis-identified in the previous paper [12].

Thus, our samples, *Proechimys* gr. *goeldii* with 2n = 15 [12], and *Proechimys* sp3 [5] all have the same multiple sex chromosome determination system, similar diploid numbers, and consistent chromosomal morphologies and G-banding patterns, suggesting that they belong to the same taxon. However, their karyotypes differ with respect to other members of the *longicaudatus* group (2n = 28), indicating that the low-diploid-number specimens belong to a distinct species. It seems likely that any progeny resulting from a mating of individuals with 2n = 14-17 and 2n = 28-30 would have many meiotic problems and could be sterile hybrids with negative heterosis. Thus, the observed chromosomal differences could indicate reproductive isolation (King, 1983). Future analyses of additional specimens will be needed to confirm if the 2n = 14-17 specimens belong to one of the nominal taxa currently considered to be synonyms of *P. brevicauda* or *P. longicaudatus* (e.g., *bolivianus*, *elassopus*, *gularis*, *leucomystax*, *ribeiroi*, *securus*, and *villacauda*[2]), or if they belong to a form that has not yet been formally described.

## Conclusions

The karyotypes described in the present paper differ from those generally accepted as representative of *P. longicaudatus*. Comparison of our karyotypes with those previously reported in the literature revealed that *Proechimys* with similar karyotypes had previously been collected from nearby localities. We thus propose that this *Proechimys* belongs to a different taxon, and is either a new species or a known species that should be reassessed.

## Competing interests

In the past five years, we have not received any reimbursement, fee, funding, or salary from an organization that could gain or lose financially from the publication of this manuscript, either now or in the future. We do not hold any stocks or shares in such organizations. We do not hold nor are we currently applying for any patents relating to the contents of this manuscript. We do not have any other financial competing interests to declare. Finally, there are no non-financial competing interests (political, personal, religious, ideological, academic, intellectual, commercial, etc.) to declare in relation to this manuscript.

## Authors’ contributions

PJSA collected some of the samples, collaborated in all of the cytogenetic procedures, undertook the bibliographic review, and coordinated the writing of this paper. CYN helped conceive the study and participated in developing the laboratory techniques, performing the cytogenetic analyses, and writing the paper. RVR and ACMO undertook the morphological analyses and discussed the data. MJRC participated in collecting the samples and developing the laboratory techniques. RCRN and MJRC performed the meiotic analysis. ALP helped with collecting samples and collaborated in the cytogenetic procedures. JCP coordinated the study, helped with developing the laboratory techniques, participated in the cytogenetic analyses, and reviewed the manuscript. All authors read and approved the final manuscript.
